# Referral, monitoring, and factors associated with non-referral of chronic kidney disease in Germany: a nationwide, retrospective cohort study

**DOI:** 10.1016/j.lanepe.2024.101111

**Published:** 2024-10-31

**Authors:** Friedrich A. von Samson-Himmelstjerna, Edgar Steiger, Benedikt Kolbrink, Hauke S. Wülfrath, Thomas Czihal, Roland Schmitt, Dominik von Stillfried, Kevin Schulte

**Affiliations:** a Department of Nephrology and Hypertension, University Hospital Schleswig-Holstein, Kiel, Germany; b Central Research Institute of Ambulatory Health Care in Germany, Berlin, Germany

**Keywords:** Chronic kidney disease, CKD diagnosis, Screening, Referral, Monitoring, Health equity, Health policy

## Abstract

**Background:**

Chronic kidney disease (CKD) is one of the most significant drivers of the global burden of disease and an increasing public health issue. Adequate monitoring and referral of high-risk patients to nephrologists are associated with improved management of CKD. We aimed to assess nephrology referral rates, monitoring of kidney function, and factors associated with failure to refer in Germany.

**Methods:**

We retrospectively analyzed ambulatory claims data of 73,675,956 German patients who were covered by statutory health care in 2022, building a cohort of 1,301,122 patients who had at least two diagnoses of CKD stage 3–5 within the calendar year. In our analysis, we focused particularly on patients with CKD stage 4.

**Findings:**

We identified 207,043 patients with CKD stage 4, of which 134,143/207,043 (64.8%) received nephrologist treatment in 2022. The median age of the cohort was 82 years. Failure to quantify proteinuria occurred in 61,991/72,900 (85.0%) non-referred patients compared to 51,382/134,143 (38.3%) referred patients. In a mixed logistic regression model, referral was less likely for women (odds ratio [OR] 0.72, 95% confidence interval [CI] 0.71–0.74), higher age (OR per year 0.97, CI 0.96–0.97), nursing home inhabitants (OR 0.63, CI 0.61–0.65), and those with certain comorbidities. Regional factors (deprivation, population density, nephrologist density) were not associated with referral.

**Interpretation:**

A substantial proportion of patients with late-stage CKD are not receiving guideline-recommended kidney care in the German health care system, with disparities driven primarily by individual patient factors rather than geographical barriers.

**Funding:**

This study was funded by the University Hospital Schleswig-Holstein and the Central Research Institute of Ambulatory Health Care in Germany.

**Research in context**

**Evidence before this study**

Chronic kidney disease (CKD) is one of the most significant contributors to the global burden of disease. Referral of patients with CKD to nephrologists is associated with improved outcomes of CKD-related morbidity and kidney failure. Adequate monitoring is essential to detecting and preventing disease progression. The degree to which the Kidney Disease: Improving Global Outcomes (KDIGO) criteria for referral and monitoring are implemented in routine care is uncertain. We identified 353 articles dated between January 1, 2005 and April 1, 2024 in a PubMed search using the terms ‘nephrology referral [Title/Abstract] OR nephrology consultation [Title/Abstract] OR kidney disease referral [Title/Abstract]’, ‘monitoring [Title] AND albuminuria [Title]’ as well as ‘monitoring [Title] AND proteinuria [Title]’. Six articles with large CKD cohorts (n > 10,000) were identified that reported on nephrology referral rates of CKD patients, factors associated with non-referral, or monitoring practice. Four of these articles reported on Northern American patients, while two studies reported on patients from regions in Sweden and Denmark. No studies were found reporting data on a national level.

**Added value of this study**

Comprehensive nationwide data adjusted for regional variances (such as population density or nephrologist density) and other variables were previously unavailable on the referral and monitoring of patients with CKD. This study analyzed current data covering approximately 87% of the German population and identified 1,301,122 patients diagnosed with CKD stages 3–5, leveraging the largest-ever European CKD cohort. Through the application of a mixed model approach, the outcomes were adjusted for both regional and individual variables. By including all claims data of the statutory health care system, this study provides an encompassing report on the sub-optimal situation of CKD management in the largest member of the European Union. While there is adequate referral for the majority of diagnosed CKD patients, a substantial proportion of patients is not referred to nephrologists, and these patients receive particularly insufficient monitoring of kidney function and proteinuria. Multi-variable adjustment showed predominantly low referral rates for women, the elderly, nursing home inhabitants, and patients with psychiatric disorders. Importantly, regional factors such as deprivation and population density had a negligible effect, indicating equitable access to nephrology care across different geographic areas. These findings reveal disparities in access to specialist care in Germany, driven by patient characteristics rather than geography.

**Implications of all the available evidence**

As the population ages, public health systems will face the challenge of managing an increasing burden of CKD-related diseases with a shrinking pool of resources. This is particularly threatening to patients with kidney failure whose lives depend on kidney replacement therapy. A proactive public health response should prioritize systemic measures aimed at preventing or delaying disease progression. The evidence found in this study shows that current screening, monitoring and referral practices do not systematically address the needs of CKD patients, indicating sub-optimal prevention of progressive CKD in Germany. This implies a need for structured measures to improve care for all patients with CKD. Furthermore, the failure to meet current guideline recommendations, despite widespread underdiagnosis of CKD, raises questions about the practicality of these guidelines in real-world settings. To address these challenges, healthcare systems must not only reinforce existing guidelines but also adapt them to ensure they are both achievable and impactful for all CKD patients.

## Introduction

Given its global prevalence of 9.1%, chronic kidney disease (CKD) is one of the most substantial contributors to the global burden of disease, and its all-age prevalence has increased by 29.3% over the past three decades.^1^ The implications for individuals are enormous, as CKD complications take a significant toll on the quality of life and life expectancy.^1^^,^^2^ In high-income countries, CKD-related spending exceeds public health care costs for heart failure treatments, yet in contrast to cardiovascular disease, stroke, and respiratory disease, CKD mortality has been rising.^3^, ^4^, ^5^ The burden of CKD has worsened by the demographic shift towards an aging population, which is projected to accelerate until 2050. This shift not only increases the prevalence of CKD-related health issues, but it also presents challenges for healthcare staffing. As more healthcare workers retire, the risk of severe understaffing for the care of patients in the resource-intensive late stages of CKD will also increase.^6^^,^^7^

Managing complications and preventing progression of CKD should, therefore, become a focus of public health policies.^8^ The pharmaceutical portfolio for the treatment of CKD has considerably grown lately, as renin-angiotensin system inhibitors,^9^ sodium-glucose cotransporter-2 inhibitors,^10^ and (in diabetic patients) glucagon-like peptide-1 receptor agonists^11^ and mineralocorticoid receptor antagonists^12^ significantly halted the progression of CKD in prospective randomized trials. A timely consultation with a nephrologist may slow disease progression,^13^ whereas late nephrology referral is associated with poor pre-kidney failure (KF) management^13^, ^14^, ^15^ as well as increased mortality.^14^, ^15^, ^16^ Therefore, the Kidney Disease: Improving Global Outcomes (KDIGO) guidelines recommend referral and frequent monitoring for those patients with highest risk for CKD progression.^2^ The goals of early identification and referral to specialist kidney care services include ensuring a specific diagnosis for CKD, providing targeted therapy, slowing disease progression, and managing comorbid conditions. This approach also involves planning for kidney replacement therapy, offering psychosocial support, and providing conservative and palliative care options when needed.^2^

Despite these recommendations, recent studies indicate that a large majority of CKD patients (approximately 70–95%) remain undiagnosed or unaware of their condition,^17^, ^18^, ^19^ which is linked to increased hospitalizations due to cardiovascular complications.^20^ Furthermore, in a study that investigated over 10,000 patients with incident dialysis in Germany, adequate dialysis preparation was implemented for only 21% of them before the initiation of dialysis.^21^ These findings suggest that even in countries with the highest healthcare expenditures per capita in the world,^22^ there is a systematic failure to detect and adequately refer patients with CKD to nephrologists.

Addressing this failure by implementing CKD-directed public health measures requires a precise understanding of current referral practices. Evidence from Northern America points towards deficiencies: only 37.8% of US veterans with prevalent CKD stage 4 were treated by nephrologists,^23^ whereas referral rates for prevalent CKD stage 4 patients were 63.9% in a US primary care cohort, with non-referral indicating lower albuminuria monitoring rates.^24^ Assessing the incident referral situation, 24.2% of US veterans^25^ and 32.6% of a Canadian primary care cohort^26^ received referral to nephrologists. The European situation appears similarly bleak: in the Stockholm CREatinine Measurements (SCREAM) study, just 9.5% of female and 16.4% of male patients with laboratory tests indicating CKD stage 4 visited a nephrologist, and similar findings emerged from a regional analysis of Southern Denmark.^18^^,^^27^ However, there are no nationwide European analyses on the prevalent referral and monitoring situation of diagnosed CKD patients. Structured programs for the comprehensive evaluation of CKD and kidney replacement therapy (KRT) are scarce in Germany, one of the few high-income countries without a structured KRT registry. In this retrospective study, we aimed to assess nephrology referral rates, monitoring of kidney function, and factors associated with failure to refer in Germany. We placed emphasis on patients with CKD stage 4, who are KDIGO-recommended to receive nephrology consultation because of their high risk for CKD-related morbidity and progression to KF (Table S1).

## Methods

### Data source

Health care coverage is required by law for German citizens. Most people receive statutory (“public”) health insurance (∼87% of the German population), while others are insured through private health care insurers. This distinction is based on factors such as income, profession, and personal choice, with higher earners and certain professionals often opting for private health insurance.

The nationwide data set used in this study contains claims data of ambulatory consultations of any kind (outside of hospitals) of 73,675,956 patients^28^ (including children) under statutory health insurance in Germany. They are routinely collected for reimbursement and can be used for scientific research according to German law (§ 295 of the Social Code Book V). The data source contains claims data available on a pseudonymized patient level for each quarter of the year, which encompasses information about age, reported sex, place of residence (county), and diagnoses according to the International Statistical Classification Of Diseases and Related Health Problems, 10th revision, German Modification (ICD-10-GM).^29^ It also contains data on procedures performed in accordance with the German fee schedule for physicians (*Gebührenordnungspositionen,* GOP).^30^

### Study period

The study included data from the years 2020–2023. The data from 2022 were used to define the study cohort (demographics, procedures, and diagnosis codes). Data from 2020 to 2022 were used to identify nephrologists and referral to nephrologists (procedures and specialist designations). Data from 2023 (procedures and any claims) were used to identify patients that were alive all through 2022, as well as to assess incidences of dialysis and palliative care.

### Outcomes of interest

The key outcomes of this study were (i) ‘nephrology referral in 2022’ and (ii) ‘monitoring of glomerular filtration rate (GFR) and proteinuria’. Nephrology referral was defined as ambulatory treatment by a physician with the specialist designation of ‘nephrologist’ or by a physician practicing dialysis in an ambulatory setting in 2022. To provide additional depth to the analysis, we also reported on the rate of patients who received care by an ambulatory nephrologist in more than one quarter of 2022 as well as on those who received care by an ambulatory nephrologist at least once in the period from 2020 to 2022. ‘Monitoring of GFR and proteinuria’ was measured by using the number of quarters in 2022 in which patients received serum-creatinine measurements (indicating estimation of the GFR [eGFR]) and/or urinalysis. The guideline-recommended formula for the eGFR in Germany is the 2009 CKD-EPI formula.^31^ Urinalysis was defined as a composite of an urine strip test, urine albumin-creatinine-ratio, or quantification of the total proteinuria. We also reported on the rates of proteinuria quantification, which included measurements of the urine albumin-creatinine-ratio and/or quantification of the total proteinuria, but not the urine strip test.

In a supplementary cohort (specified below), further outcomes of interest were the uncensored 2-year incidences of ambulatory dialysis or ambulatory palliative care. These outcomes were treated as binary variables and measured within a 2-year observation period between the first quarter in 2022 and the last quarter of 2023.

### Exclusion criteria

To ensure data plausibility, the study cohort was restricted to age <125 years, with reported sex specified as female or male, and with available information on the region of residence (Figure S1). To provide a conservative estimation of the non-referral rate, and in order to be able to compare monitoring rates in our study to the KDIGO recommendations for times of monitoring per year, patients who exited the statutory health system during 2022 (e.g. due to death, switching to private health insurance, or moving abroad) were excluded from the main analyses. Since these reasons are not explicitly detailed in the data source, we achieved this by including only patients who had at least one ambulatory health claim between the last quarter of 2022 and the last quarter of 2023 (n = 69,050,132). This sample from the 2022 general patient population was the reference cohort for calculating CKD prevalence in our study.

### Study cohort and definition of CKD

We built a CKD cohort based on a reconfirmed diagnosis of CKD stages 3–5 in the year 2022 according to the M2Q criterion.^32^ This means that a diagnosis of CKD in 2022 was considered as reconfirmed when it was followed up by a second diagnosis of CKD in at least one additional quarter in 2022. CKD was defined as a claimed diagnosis of stage 3 (N18.3), stage 4 (N18.4), stage 5 (N18.5), kidney transplantation (Z94.0), and/or a dialysis session (hemodialysis or peritoneal dialysis). It should be noted that the ICD-10-GM code for stage 3 did not allow for the sub-specification of CKD stage 3a vs. the more advanced CKD stage 3b, and that an ICD-10 code for KF (N18.6) does not exist in the German modification. Each patient with CKD was assigned a CKD stage (Table S1), as specified in the Supplementary Material. KRT was defined as receiving any form of kidney replacement therapy, including hemodialysis, peritoneal dialysis, or kidney transplantation.

We also created a supplementary cohort to evaluate the progression of CKD stage 4 patients to either ambulatory dialysis or palliative care within a two-year observation period. For this, we selected patients diagnosed with CKD stage 4 in the first quarter of 2022, with the diagnosis reconfirmed in another quarter of 2022. This was done regardless of whether the patients exited the statutory health care system in 2022 or 2023 (see Figure S1).

### Covariates

We extracted the reported sex and age of patients within the 2022 cohort. From the claims data, we identified nursing home inhabitants as well as patients receiving palliative care in 2022. We used the Elixhauser index to classify comorbidities, following a hierarchical approach where more complex conditions took precedence over less complex ones. All Elixhauser comorbidities (amongst others, diabetes mellitus and arterial hypertension) were identified using ICD-10-GM codes. Population density, nephrologist density, and deprivation scores^33^ were categorized into deciles, each representing 10% of the population, from lowest to highest. Further details on the extraction of the covariates are provided in the Supplementary Material.

Continuous covariates (age, population density, nephrologist density, deprivation score, and Elixhauser score) were characterized by median (50th percentile) and interquartile range (25th and 75th percentile), nephrology referral and other categorical (binary) covariates (sex, nursing home inhabitation, palliative care, diabetes mellitus, and arterial hypertension) were characterized by count and percentage of total. As sub-cohorts, we defined age brackets by sex (0–30, 31–50, 51–70, 71–85, and 86 or more years, female and male respectively).

### Statistical analysis

All statistical analyses were conducted using the R statistical programming language.^34^ References for all the packages are provided in the Supplementary Material. All p values < 0.05 were considered statistically significant. We analyzed the referral rates of patients in different CKD stages to nephrologists. To adjust for factors confounding the outcome ‘referral to a nephrologist in 2022’, we applied a mixed logistic regression model with the independent variables of age, sex, nursing home inhabitant status, palliative care, Elixhauser score (fixed effects at the individual level); deprivation index, population density, nephrologist density (fixed effects at the regional level); as well as county and state (random effects at the regional level). The resulting coefficients are presented as odds ratios (exponentiated estimates, OR) with their 95-percent confidence intervals and p-values. The impact of the random factors (county and state levels) was assessed using the intra-class correlation coefficient (ICC). The same mixed logistic regression model was applied in a sensitivity analysis of CKD stage 3 and CKD stage 5 without KRT sub-cohorts (Supplementary Material).

### Role of the funding source

Neither the University Hospital Schleswig–Holstein nor the Central Research Institute had a role in the study design, collection, analysis, interpretation of the data, writing the report or decision to submit the paper for publication.

## Results

### Prevalence of referral and monitoring in CKD patients

**Fig. 1:**
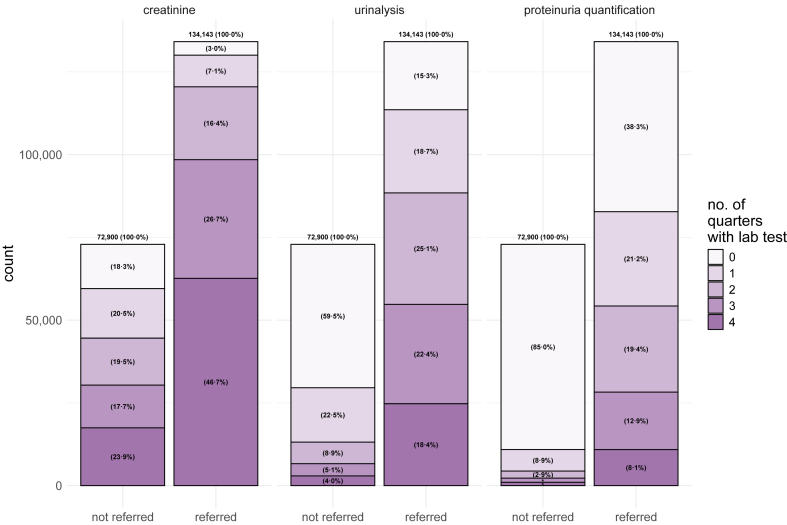
**Frequency of serum creatinine and proteinuria monitoring in patients with CKD stage 4, comparing those referred and not referred to a nephrologist.** The figure shows the number of quarters in 2022 during which serum creatinine was measured, any type of urinalysis was conducted, and total proteinuria was quantified. Patients are grouped by referral status, with referral defined as having at least one ambulatory health claim from a nephrologist in 2022. Percentages (%) represent the proportion of patients in each referral group, relative to the total number of patients in that group. *n* (total) = 207,043. CKD, chronic kidney disease. **∗** proteinuria quantification, not referred: 3 quarters (1.7%), 4 quarters (1.4%).

We detected 958,149 patients with CKD stage 3 (prevalence 1.4%), 207,043 patients with CKD stage 4 (prevalence 0.3%), 37,020 patients with CKD stage 5 without KRT (prevalence 0.1%), and 98,910 patients with CKD stage 5 with KRT (prevalence 0.1%), adding up to a total of 1,301,122 patients (prevalence 1.9%) with CKD stages 3–5 (Table S2). Referral to nephrology was 35.9% in patients with CKD stage 3 (Table 1). The referral rates for CKD stage 4 and stage 5 without KRT reached 64.8% and 74.6%. The vast majority of transplanted patients (92.5%) and all patients receiving dialysis were referred (100.0%). We analyzed the sub-cohort with CKD stage 4 more closely. The majority of CKD stage 4 patients were female (56.6%) and the median age was 82 years (Table 2). Diabetes mellitus (58.8%) and arterial hypertension (95.4%) were common comorbidities. During the three-year window from 2020 to 2022, 76.4% had received ambulatory nephrology care (Table 1). One third of CKD stage 4 patients were seen by a nephrologist in three or more different quarters in 2022 (33.6%). Out of 72,900 non-referred patients, 13,366 (18.3%) did not receive serum creatinine monitoring, 43,341 (59.5%) did not undergo any urinalysis, and 61,991 (85.0%) did not have proteinuria quantification (Fig. 1). Among 134,143 referred patients, 4082 (3.0%) did not receive serum creatinine monitoring, 20,580 (15.3%) did not undergo any urinalysis, and 51,382 (38.3%) did not have proteinuria quantification. Further analyses revealed that also in patients with CKD stage 3 and CKD stage 5 without KRT, kidney function monitoring occurred more frequently among those referred to nephrologists (Figures S2 and S3).

**Table 1:** Frequency of nephrology referral in Germany. Patients with a reconfirmed diagnosis of CKD stage 3–5 in the year 2022 were included in this analysis.

CKD stage	Count	Referral to a nephrologist …									
		in 2022								at least once in 2020–2022	
		One or more quarters		Two or more quarters		Three or more quarters		Four or more quarters			
3	958,149	344,358	35.9%	187,009	19.5%	86,966	9.1%	38,164	4.0%	494,449	51.6%
4	207,043	134,143	64.8%	106,097	51.2%	69,649	33.6%	38,028	18.4%	158,241	76.4%
5—without KRT	37,020	27,617	74.6%	24,632	66.5%	20,834	56.3%	16,400	44.3%	30,560	82.5%
5—with KRT	98,910	96,737	97.8%	95,640	96.7%	93,050	94.1%	88,096	89.1%	97,557	98.6%
dialysis	64,722	64,722	100%	64,515	99.7%	62,891	97.2%	60,141	92.9%	64,722	100%
KTx	29,017	26,844	92.5%	25,977	89.5%	25,065	86.4%	22,996	79.3%	27,664	95.3%
KTx + dialysis	5171	5171	100%	5148	99.6%	5094	98.5%	4959	95.9%	5171	100%
Total	1,301,122	602,855	46.3%	413,378	31.8%	270,499	20.8%	180,688	13.9%	780,807	60.0%

This table reports on the absolute count and the proportion of CKD patients who visited a nephrologist in at least one, at least two, at least three or at least four quarters in 2022 (central four columns). Additionally, the absolute count and proportion of patients who saw a nephrologist at least once between 2020 and 2022 is reported (far right column). Referral to a nephrologist was defined as a health claim by an ambulatory nephrologist. KTx patients were counted as ‘CKD stage 5 with KRT’, but if graft failure was present, they were identified as ‘KTx + dialysis’ in the table. CKD, chronic kidney disease; KRT, kidney replacement therapy; KTx, kidney transplantation.

**Table 2:** Baseline characteristics of patients with CKD stage 4.

	Total	Not referred	Referred
Count	207,043	72,900	134,143
Age in y, median (IQR)	82 (73–86)	84 (77–88)	81 (72–85)
Age brackets, count (%)			
0–30 years	752 (0.4%)	116 (0.2%)	636 (0.5%)
31–50 years	4140 (2.0%)	807 (1.1%)	3333 (2.5%)
51–70 years	34,343 (16.6%)	9010 (12.4%)	25,333 (18.9%)
71–85 years	106,938 (51.7%)	33,866 (46.5%)	73,072 (54.5%)
86+ years	60,870 (29.4%)	29,101 (39.9%)	31,769 (23.7%)
Female sex, count (%)	117,134 (56.6%)	46,229 (63.4%)	70,905 (52.9%)
Nursing home inhabitant, count (%)	20,341 (9.8%)	10,187 (14.0%)	10,154 (7.6%)
Palliative care in 2022, count (%)	5789 (2.8%)	2656 (3.6%)	3133 (2.3%)
Population density, decile median (IQR)	5 (2–7)	4 (2–7)	5 (2–7)
Deprivation index, decile median (IQR)	6 (4–8)	6 (3–8)	6 (4–8)
Nephrologist density, decile median (IQR)	5 (3–7)	5 (2–7)	5 (3–7)
Elixhauser index, median (IQR)	7 (5–8)	6 (5–8)	7 (5–9)
Diabetes mellitus, count (%)	121,754 (58.8%)	42,193 (57.9%)	79,561 (59.3%)
Arterial hypertension, count (%)	197,594 (95.4%)	67,689 (92.9%)	129,905 (96.8%)

Referred patients had at least one ambulatory health claim by a nephrologist in 2022. Continuous and ordinal data are presented as medians with the interquartile range (25th and 75th quartiles). Binary and categorical data are presented as counts with the proportion of the total population (in %). The German population was sorted by deciles to analyze the factors of population density, deprivation index, and nephrologist density. Decile 10 indicates a patient belonging to the decile of the German population with the highest population density/highest deprivation/highest nephrologist density. Conversely, decile 1 indicates a patient belonging to the decile of the German population with the lowest population density/lowest deprivation/lowest nephrologist density. CKD, chronic kidney disease; IQR, interquartile range; y, year.

### Variables associated with referral

**Fig. 2:**
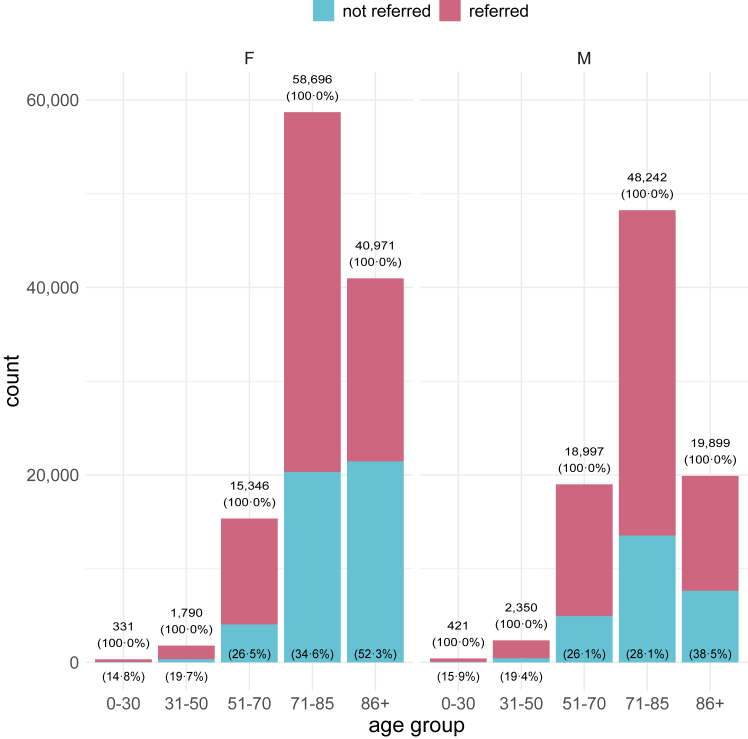
**Age-dependent referral patterns for males and females with CKD stage 4 to nephrology services.** Patients were stratified by reported sex (male and female) and grouped by age ranges. Referral was defined by the presence of at least one ambulatory health claim from a nephrologist in 2022. The figure displays the percentage of non-referred patients in each age group, with the remaining percentage (not explicitly shown) representing referred patients. The sum of these proportions equals 100% for each age group. *n* (total) = 207,043. CKD, chronic kidney disease; F, female; M, male.

We analyzed the relationships between age, reported sex, and nephrology referral by stratifying CKD stage 4 patients (Fig. 2). Among younger patients aged 31–50 years, 455 out of 2350 (19.4%) males and 352 out of 1790 (19.7%) females were not referred to a nephrologist. As age increased, non-referral rates also rose, with a widening gap between males and females. In patients aged 86 years and older, 7653 out of 19,899 (38.5%) males and 21,448 out of 40,971 (52.3%) females were not referred. These same patterns were found in the patients with CKD stage 3 or stage 5 without KRT (Figures S4 and S5, respectively). Notably, among CKD stage 3 patients aged 31–50 years, 3826 out of 9273 (41.3%) males and 2775 out of 7092 (39.1%) females were not referred.

To verify these findings in the adjusted analyses, we analyzed factors associated with referral using a multi-variable mixed logistic regression model. The ICCs for county and state were 0.043 and 0.012, respectively (Table 3). While higher morbidity was associated with nephrology referral (OR per Elixhauser point 1.14, CI 1.14–1.15), the variables age (OR per year 0.97, CI 0.96–0.97), female sex (OR 0.72, CI 0.71–0.74), nursing home inhabitant (OR 0.63, CI 0.61–0.65) and palliative care (OR 0.80, CI 0.76–0.85) were strongly associated with non-referral. Deprivation (OR per decile 1.02, CI 1.00–1.04), population density (OR per decile 1.00, CI 0.99–1.02) and nephrologist density (OR per decile 1.00, CI 0.99–1.01) were not associated with nephrology referral in the adjusted analyses, while they were positively associated with referral in the unadjusted analyses. A sensitivity analysis using all Elixhauser comorbidities as individual variables did not change the associations of the variables as described above (Table S3). Several individual comorbidities were negatively or positively associated with referral in the adjusted analyses, with psychiatric diagnoses (alcohol abuse, drug abuse, psychosis and depression) being uniformly associated with non-referral. Further sensitivity analyses of patients with CKD stage 3 and stage 5 without KRT revealed findings similar to those found in CKD stage 4 patients (Tables S4 and S5). The most notable deviance was seen in nursing home inhabitants and palliative care recipients, who had increased odds of being referred in CKD stage 3.

**Table 3:** Determinants of nephrology referral in CKD stage 4.

Random effect	Intra-class coefficient
County	0.043
State	0.012

Fixed effect	Unadjusted OR	CI	p-value	Adjusted OR	CI	p-value
(Intercept)	–	–	–	13.79	(11.10–17.13)	(<0.00001)
Female sex	0.65	0.64–0.66	<0.00001	0.72	0.71–0.74	<0.00001
Age per year	0.97	0.96–0.97	<0.00001	0.97	0.96–0.97	<0.00001
Nursing home inhabitant	0.50	0.49–0.52	<0.00001	0.63	0.61–0.65	<0.00001
Palliative care	0.63	0.60–0.67	<0.00001	0.80	0.76–0.85	<0.00001
Deprivation score, per decile	1.04	1.03–1.04	<0.00001	1.02	1.00–1.04	0.051
Population density, per decile	1.01	1.00–1.01	<0.00001	1.00	0.99–1.02	0.747
Nephrologist density, per decile	1.01	1.01–1.01	<0.00001	1.00	0.99–1.01	0.932
Elixhauser score, per point	1.11	1.11–1.11	<0.00001	1.14	1.14–1.15	<0.00001

A multi-variable mixed logistic regression model was used to evaluate nephrologist referral in 2022. Referred patients had at least one health claim by a nephrologist. The intra-class coefficient (ICC) measures the proportion of total variance attributable to county and state levels, with values closer to 1 indicating more variation between regions and values closer to 0 indicating more variation between individuals. Female sex, nursing home inhabitant, and palliative care were binary variables; age was continuous. Deprivation score, population density, and nephrologist density were categorized into deciles to assess their impact across the distribution of the German population, with higher deciles indicating higher density or deprivation. Higher Elixhauser scores indicated greater comorbidity. *n*(total) = 207,043; *n*(endpoint) = 134,143. CKD, chronic kidney disease; CI, confidence interval; OR, odds ratio.

To assess the risk for disease progression in different age groups, we further evaluated the uncensored 2-year incidence of prevalent CKD stage 4 patients to receive ambulatory palliative care or ambulatory dialysis in a sub-cohort (Table S6). This analysis included 160,965 patients with CKD stage 4 in the first quarter of 2022, regardless of whether they subsequently exited the statutory health care system. The incidence of palliative care was slightly higher in non-referred patients than in referred patients, continuously increasing by age, and reaching up to 10.7% in those age 86 years or older. Conversely, the uncensored 2-year incidence of ambulatory dialysis was much higher in referred patients compared to non-referred patients, peaking at 14.3% in patients aged 0–30 years and continuously decreasing with higher age.

## Discussion

Our study has two major findings: i) the referral and monitoring of CKD patients in Germany occur less frequently than recommended by international guidelines, and ii) while certain patient groups face disparities in care, we observed equitable access to nephrology services across geographic regions.

The current demographic changes imply that a shrinking pool of adequately qualified human resources will be available to manage the growing burden of disease. CKD is a highly prevalent disease, and the disease-associated costs increase with each disease stage.^4^ Considering these circumstances, preventing the progression of early CKD stages to KF and timely management of CKD-related complications are critical tasks. However, to achieve these goals, CKD must be diagnosed, and patients at high risk for CKD progression require referral to specialists as well as sufficient monitoring of kidney function. Our study shows that these requirements are not sufficiently met in Germany. Despite including very old patients who have a particularly high CKD prevalence, the diagnosed disease prevalence (in other words: recorded disease prevalence) of CKD stages 3–5 in our study was just 1.9%. This lies well below previously reported prevalences between 3.1% and 5.9% in cross-sectional studies that had even excluded patients older than 82 years.^35^ Several recent studies support this observation, highlighting the substantial rates of underdiagnosis of CKD in Germany and other high-income countries.^17^ Considering that already among the diagnosed CKD stage 4 patients in our study one third had not been referred, it is reasonable to assume that the true percentage of patients in Germany who are not referred despite having CKD stage 4 is high. This fails to meet the clear recommendations set by the KDIGO guidelines.^2^ Additionally, the monitoring frequency of kidney function did not meet the goals suggested by the guidelines (at least three times per year) either, particularly for those patients not referred to nephrologists, but it should be noted that the evidence that these suggestions were based on is less substantive.^2^

Our study identified significantly lower nephrology referral rates due to individual patient factors, while regional influences (county, state) appeared negligible. Consistent with prior research from the Stockholm region,^18^ women were less likely to receive nephrology care than men, even after multiple adjustments, highlighting the need to investigate this sex bias globally. While referral rates for young patients were far from perfect, we found very low referral rates among elderly patients—particularly women—and nursing home inhabitants, in line with evidence from a meta-analysis showing delayed referral in these groups.^36^ The uncensored 2-year dialysis incidence was relatively low for very elderly patients in this study, and some argue that nephrology referral should prioritize younger patients.^37^ However, in a previous study, we showed that more than a quarter of incident dialysis patients in Germany were ≥80 years old, many of which did not survive the initial hospital stay, underscoring the significant absolute number of elderly dialysis patients on a population-wide scale.^21^ Along these lines, the present study suggests that selective referral of elderly patients with a better overall health status is practiced in Germany, as referred patients were less likely to need palliative care but had a higher chance of starting KRT. Apart from receiving KRT, the elderly may benefit from discussing palliative care options with a nephrologist, or from other advantages of a nephrology referral such as the management of CKD-specific diseases (bone disease, malnutrition, and anemia) or cardiovascular disease.^2^^,^^14^ Thus, age should not be a principal barrier to specialist care. Our data also suggest lower referral rates for patients with psychiatric disorders, which is particularly worrisome because dosing of psychotropic medication becomes increasingly difficult as kidney function declines, psychotropic drugs such as lithium may further perpetuate kidney disease, and CKD patients report increased levels of psychiatric symptoms such as anxiety, insomnia, or depression.^38^

On a positive note, the adjusted analyses revealed that patients living in deprived or less populated geographical areas did not face restricted access to nephrology care, highlighting a favorable aspect of the German health care system. Moreover, referral of prevalent patients with CKD stage 4 was similarly high in our study as in a US primary care cohort.^24^

Referral and monitoring rates of CKD are already lower than recommended among diagnosed CKD patients in Germany. With millions more undiagnosed, it is not feasible for all CKD patients to frequently receive specialized nephrology care. Instead, diagnosed cases of kidney disease due to diabetes mellitus or hypertension probably do not need to see a nephrologist more than once a year, and in stable disease, monitoring should be safely possible through primary care. Currently, nephrologists in Germany are disproportionally poorly compensated for providing preventative and palliative care services compared to providing dialysis. To prepare the health care system for the rising burden of kidney disease in years to come, we suggest implementation of public health actions in Germany that address CKD screening in populations at risk, prevention of CKD progression, and improved management of patients with (pre-)KF. The success of similar approaches has previously been demonstrated by others, e.g. in the CKD management program of the National Health Service in Birmingham^39^ and the Taiwanese CKD care program.^40^

Several strengths and limitations of this study should be considered. The data source contained all ambulatory claims data of all German statutory health care recipients, thus representing a very large patient cohort of approximately 87% of the German population. However, data from treatment in hospitals (e.g. inpatient urinalysis or inpatient dialysis) was not contained in the data source and we had no data on patients with private health insurance (approximately 13% of the German population). Importantly, the data had not primarily been collected for research purposes but for the purpose of reimbursement. While the diagnosis of CKD in German Health claims has very robust specificity for the underlying condition,^41^ the data were consequently biased to contain only CKD patients who had been diagnosed. Undiagnosed CKD patients would be better captured by a cross-sectional approach, as seen in the SCREAM study.^18^ The data source restricted our ability to adjust the analyses for laboratory data such as parameters of renal function (e.g. GFR or proteinuria), and we were not able to further stratify the many unreferred CKD stage 3 patients more closely in terms of their KDIGO-recommended indication for referral. We used a mixed model approach to reduce problems associated with factors that were available only at the regional level (e.g. ecological fallacy). Furthermore, our findings are representative for the German population, but they may differ from situations in other parts of the world. Last, all data contained in this study were analyzed retrospectively. Therefore, we did not attempt to causally explain the impact of nephrology referral on treatment outcomes.

The progression of CKD and CKD-related complications are highly significant contributors to the global burden of disease and can be managed efficiently. In this nationwide study, we identified vulnerabilities in key aspects of CKD management, calling for installation of public health policies directed at the identification and appropriate management of patients suffering from CKD.

## Contributors

Conceptualization: all authors, data curation: FAvSH and ES, formal analysis: FAvSH and ES, funding acquisition: DvS and KS, investigation: all authors, methodology: FAvSH, ES and BK, project administration: TC, RS, DvS and KS, resources: TC, RS, DvS and KS, software: ES, supervision: DvS and KS, validation: FAvSH and ES, visualization: FAvSH and ES, writing—original draft: FAvSH and ES, and writing—review & editing: BK, HSW, TC, RS, DvS and KS.

## Data sharing statement

The claims data analyzed during this study are not publicly available due to the regulations for sensitive health data in Article 9 of the General Data Protection Regulation (GDPR) of the European Union. Limited access can be given within the context of specific research projects and following regulations of the German Social Code Book. The authors are available to discuss such possibilities.

## Declaration of interests

FAvSH reports receiving lecturing fees from AstraZeneca and travel support from AstraZeneca and Chiesi GmbH. BK reports research grants to his institution from Baxter, Fresenius Medical Care, Sanofi, and Astellas. HW reports receiving travel support from Amgen and AstraZeneca, receiving financial compensation for participating in an advisory board for Novartis, and receiving equipment from Fresenius Medical Care and Baxter for educational purposes (to institution). ES, TC, RS, DvS, and KS report no conflict of interest.

The Central Research Institute is a foundation primarily funded by the 17 Associations of Statutory Health Insurance Physicians in Germany which are mandated to guarantee equitable access to statutory ambulatory medical care. Its task is to provide research in support of the implementation of this mandate.

## Appendix A Supplementary data

German abstract
