# Supplementary material for: Referral, monitoring, and factors associated with non-referral of chronic kidney disease in Germany: a nationwide, retrospective cohort study
Source: Lancet Reg Health Eur. 2024 Oct 31;47:101111. doi: 10.1016/j.lanepe.2024.101111 (PMC11670680; doi:10.1016/j.lanepe.2024.101111)
Supplement: German abstract [file mmc1.docx]

# German Abstract

**Hintergrund**
Die chronische Nierenerkrankung (*chronic kidney disease*, CKD) ist ein bedeutender Faktor der globalen Krankheitslast und eine zunehmende Herausforderung für die öffentliche Gesundheit. Eine angemessene Diagnostik und Überwachung sowie die Überweisung von Hochrisikopatienten und -patientinnen in die fachärztliche Betreuung verbessert das CKD-Management. Diese Studie untersuchte das Ausmaß der Umsetzung dieser Maßnahmen in Deutschland.

**Methoden**
Wir analysierten retrospektiv Abrechnungsdaten von 73,675,956 im Jahr 2022 gesetzlich Versicherten in Deutschland, von welchen 1,301,122 mindestens zwei Diagnosen von CKD im Stadium 3-5 erhalten hatten. Der Fokus der weiteren Analysen lag auf dem CKD-Stadium 4.

**Ergebnisse**
Eine nephrologische fachärztliche Mitbetreuung erfolgte bei 134,143 von 207,043 (64·8%) Versicherten im CKD-Stadium 4. Das Alter betrug im Median 82 Jahre. Bei 61,991/72,900 (85·0%) der Nicht-Überwiesenen und bei 51,382/134,143 (38·3%) der Überwiesenen wurde keine Proteinurie-Quantifizierung durchgeführt. In einem gemischten logistischen Regressionsmodell war die fachärztliche Überweisung weniger wahrscheinlich bei Frauen (Odds Ratio [OR] 0·72, 95% Konfidenzintervall [KI] 0·71-0·74), höherem Alter (OR pro Jahr 0·97, KI 0·96-0·97), Pflegeheimbewohnenden (OR 0·63, KI 0·61-0·65) und bestimmten Komorbiditäten. Geographische Faktoren zeigten keinen Einfluss auf die Überweisungswahrscheinlichkeit.

**Interpretation**
Oftmals erfolgt trotz fortgeschrittener CKD keine leitliniengerechte nephrologische Versorgung. Unterschiede wurden hauptsächlich durch individuelle Faktoren verursacht, nicht durch geografische Hindernisse.

**Förderung**
Die Studie wurde vom Universitätsklinikum Schleswig-Holstein und dem Zentralinstitut für die kassenärztliche Versorgung finanziert.
