# Supplementary material for: Referral, monitoring, and factors associated with non-referral of chronic kidney disease in Germany: a nationwide, retrospective cohort study
Source: Lancet Reg Health Eur. 2024 Oct 31;47:101111. doi: 10.1016/j.lanepe.2024.101111 (PMC11670680; doi:10.1016/j.lanepe.2024.101111)
Supplement: Supplementary Figures and Tables [file mmc2.pdf]

Referral and monitoring of chronic kidney disease in Germany: a nationwide, retrospective cohort study

Friedrich A. von Samson-Himmelstjerna[1]*, Edgar Steiger[2]*, Benedikt Kolbrink[1], Hauke S. Wülfrath[1], Thomas Czihal[2], Roland Schmitt[1], Dominik von Stillfried[2], Kevin Schulte[1]

1) Department of Nephrology and Hypertension, University Hospital Schleswig-Holstein, Kiel, Germany

2) Central Research Institute of Ambulatory Health Care in Germany, Berlin, Germany

* These authors contributed equally.

Supplementary Material

Table of contents

Table of contents 1
Supplementary Methods 2
  Missing data 2
  Definitions and Covariates 2
  Sensitivity analyses 2
  ICD-10-GM codes 2
  GOP codes 4
  Software 4
Supplementary Tables 5
  Table S1. Overview of CKD stages and KDIGO recommendations for referral and monitoring. 5
  Table S2. Prevalence of CKD stages 3-5 and baseline characteristics. 6
  Table S3. Sensitivity analysis of table 3 including all individual comorbidities of the Elixhauser index. 8
  Table S4. Determinants of nephrology referral in CKD stage 3. 9
  Table S5. Determinants of nephrology referral in CKD stage 5 without KRT. 10
  Table S6. 2-year incidence of ambulatory dialysis and ambulatory palliative care among prevalent CKD stage 4 patients. 11
Supplementary Figures 12
  Figure S1. Flowchart of the inclusion of patients. 12
  Figure S2. Frequency of serum creatinine and proteinuria monitoring in patients with CKD stage 3, comparing those referred and not referred to a nephrologist. 13
  Figure S3. Frequency of serum creatinine and proteinuria monitoring in patients with CKD stage 5 without KRT, comparing those referred and not referred to a nephrologist. 14
  Figure S4. Age-dependent referral patterns for males and females with CKD stage 3 to nephrology services. 15
  Figure S5. Age-dependent referral patterns for males and females with CKD stage 5 without KRT to nephrology services. 16
Supplementary References 17

Supplementary Methods

Missing data

After performing a data plausibility check and selecting the cohort as outlined in figure S1 and in the exclusion criteria (see Methods), there were no overtly missing data in our data set. Generally, claims data may be prone to non-coding of disorders or procedures. While these circumstances need to be considered in the interpretation of the data (see Discussion), they did not affect our analyses computationally.

Definitions and Covariates

Diagnoses in claims data We identified diagnoses using the diagnostic claims of the International Statistical Classification of Diseases and Related Health Problems, 10th revision, German Modification (ICD-10-GM). We used diagnoses marked as ‘confirmed’ (G) whilst disregarding diagnoses marked as ‘exclusion of’ (A), ‘condition after’ (Z), and ‘suspicion of’ (V).

Nephrology referral All German statutory health insurance physicians were included in the analysis. We used the following codes of *Weiterbildungsordnung,*[1] which is the German word for “continuing medical education regulations,” to identify nephrologists: 086, 096, 526, 538, and 545. These codes all define physicians who have completed a nephrology fellowship. Physicians are identified by a pseudonymized ID, but in some cases, because of data extraction errors, cases had been assigned a physician fallback ID (6816) with an unspecified specialist designation in the data available to us. To maintain a conservative approach, we considered any case of CKD patient with a physician fallback ID to be a nephrologist referral, thereby slightly overestimating the true referral rate. All patient IDs with a claim submitted by a nephrologist in 2022 were considered to have received a nephrology referral.

Chronic kidney disease (CKD) diagnosis and M2Q The M2Q criteria for a reconfirmed CKD case require at least two confirmed (G) CKD diagnosis codes during two different quarters in 2022. The CKD stage is specified as follows using the M2Q criteria for a given patient. The most severe confirmed CKD diagnosis code in every quarter is considered the most severe code appearing twice with four possible codes that define the CKD stage. If no code appears twice, the least severe of these four possible codes defines the CKD stage.

Example 1: Q1 N18.4, Q2 ---, Q3 N18.5, Q4 N18.4 → CKD stage 4 (N18.4)

Example 2: Q1 ---, Q2 N18.2, Q3 ---, Q4 N18.3 → CKD stage 2 (N18.2)

The Elixhauser Index We used the Elixhauser Index as defined by Quan et al.[2,3] ‘Complicated diabetes’ and ’Complicated hypertension’ were considered to be higher on the hierarchical order of complexity than ‘uncomplicated diabetes/hypertension’, respectively (uncomplicated disease was not considered if complicated disease was present). Consequently, ‘metastatic cancer’ was higher on the hierarchical order than ‘solid tumor’ was.

Population density, nephrologist density, and deprivation scores. Population density, nephrologist density, and deprivation scores were grouped into deciles, that is, the population was sorted among these three variables, respectively, with the bottom 10% assigned to the first decile, the second 10% assigned to the second decile, and so on, until the top 10% was assigned to the 10th/last decile.

Comorbidities

Comorbidities were identified through the respective ICD-10-GM codes and handled as categorical data. Palliative care was identified through the respective *Gebührenordnungspositionen* (GOP) codes, which are codes for claiming health procedures (diagnostics, therapies).

Sensitivity analyses

CKD severity ranges on a continuous spectrum which was subdivided into five stages by international consensus. To substantiate findings from the CKD stage 4 sub-cohort, we added sensitivity analyses on the neighboring CKD stage 3 and 5 without kidney replacement therapy (KRT).

ICD-10-GM codes

	Diagnosis	ICD-10-GM code
**CKD stage**	CKD stage 1	N18.1
	CKD stage 2	N18.2
	CKD stage 3	N18.3
	CKD stage 4	N18.4
	CKD stage 5	N18.5
	Other CKD	N18.8, N18.89
	CKD, stage unspecified	N18.9

	Kidney transplant recipient	Z94.0
**Elixhauser Index**	Congestive heart failure	I09.9, I11.0, I13.0, I13.2, I25.5, I42.0, I42.5, I42.6, I42.7, I42.8, I42.9, I43, I50, P29.0
	Cardiac arrhythmia	I44.1, I44.2, I44.3, I45.6, I45.9, I47, I48, I49, R00.0, R00.1, R00.8, T82.1, Z45.0, Z95.0
	Valvular disease	A52.0, I05, I06, I07, I08, I09.1, I09.8, I34, I35, I36, I37, I38, I39, Q23.0, Q23.1, Q23.2, Q23.3, Z95.2, Z95.3, Z95.4
	Pulmonary circulation disorder	I26, I27, I28.0, I28.8, I28.9
	Peripheral vascular disease	I70, I71, I73.1, I73.8, I73.9, I77.1, I79.0, I79.2, K55.1, K55.8, K55.9, Z95.8, Z95.9
	Hypertension without complications	I10
	Hypertension with complications	I11, I12, I13, I15
	Paralysis	G04.1, G11.4, G80.1, G80.2, G81, G82, G83.0, G83.1, G83.2, G83.3, G83.4, G83.9
	Other neurological disorders	G10, G11, G12, G13, G20, G21, G22, G25.4, G25.5, G31.2, G31.8, G31.9, G32, G35, G36, G37, G40, G41, G93.1, G93.4, R47.0, R56
	Chronic pulmonary disease	I27.8, I27.9, J40, J41, J42, J43, J44, J45, J46, J47, J60, J61, J62, J63, J64, J65, J66, J67, J68.4, J70.1, J70.3
	Diabetes without chronic complications	E10.0, E10.1, E10.9, E11.0, E11.1, E11.9, E12.0, E12.1, E12.9, E13.0, E13.1, E13.9, E14.0, E14.1, E14.9
	Diabetes with chronic complications	E10.2, E10.3, E10.4, E10.5, E10.6, E10.7, E10.8, E11.2, E11.3, E11.4, E11.5, E11.6, E11.7, E11.8, E12.2, E12.3, E12.4, E12.5, E12.6, E12.7, E128, E13.2, E13.3, E13.4, E13.5, E13.6, E13.7, E13.8, E14.2, E14.3, E14.4, E14.5, E14.6, E14.7, E14.8
	Hypothyroidism	E00, E01, E02, E03, E89.0
	Liver disease	B18, I85, I86.4, I98.2, K70, K71.1, K71.3, K71.4, K71.5, K71.7, K72, K73, K74, K76.0, K76.2, K76.3, K76.4, K76.5, K76.6, K76.7, K76.8, K76.9, Z94.4
	Peptic ulcer disease	K25.7, K25.9, K26.7, K26.9, K27.7, K27.9, K28.7, K28.9
	Human immunodeficiency virus	B20, B21, B22, B24
	Lymphoma	C81, C82, C83, C84, C85, C88, C96, C90.0, C90.2
	Metastatic cancer	C77, C78, C79, C80
	Solid tumor without metastasis	C00, C01, C02, C03, C04, C05, C06, C07, C08, C09, C10, C11, C12, C13, C14, C15, C16, C17, C18, C19, C20, C21, C22, C23, C24, C25, C26, C30, C31, C32,

		C33, C34, C37, C38, C39, C40, C41, C43, C45, C46, C47, C48, C49, C50, C51, C52, C53, C54, C55, C56, C57, C58, C60, C61, C62, C63, C64, C65, C66, C67, C68, C69, C70, C71, C72, C73, C74, C75, C76, C97
	Rheumatoid arthritis	L94.0, L94.1, L94.3, M05, M06, M08, M12.0, M12.3, M30, M31.0, M31.1, M31.2, M31.3, M32, M33, M34, M35, M45, M46.1, M46.8, M46.9
	Coagulopathy	D65, D66, D67, D68, D69.1, D69.3, D69.4, D69.5, D69.6
	Obesity	E66
	Weight loss	E40, E41, E42, E43, E44, E45, E46, R63.4, R64
	Fluid and electrolyte disorders	E22.2, E86, E87
	Blood loss anemia	D50.0
	Anemia deficiency	D50.8, D50.9, D51, D52, D53
	Alcohol abuse	F10, E52, G62.1, I42.6, K29.2, K70.0, K70.3, K70.9, T51, Z50.2, Z71.4, Z72.1
	Drug abuse	F11, F12, F13, F14, F15, F16, F18, F19, Z71.5, Z72.2
	Psychosis	F20, F22, F23, F24, F25, F28, F29, F30.2, F31.2, F31.5
	Depression	F20.4, F31.3, F31.4, F31.5, F32, F33, F34.1, F41.2, F43.2

GOP codes

	**Procedure**	**GOP code**
**Dialysis**	Hemodialysis	40815, 40818, 40823, 40824, 40828
	Peritoneal dialysis	40816, 40817, 40819, 40825, 40826, 40827, 40837, 40838
**Palliative care**	Palliative care	03370, 03371, 03372, 03373
**Monitoring of eGFR**	Serum creatinine measurement	32067, 32066, 01930
**Urinalysis**	Urine test strip	32033, 32880
	Urinary creatinine-albumin ratio	32135
	Quantification of total protein (urine)	32237
**Quantification of proteinuria**	Urinary creatinine-albumin ratio, micro-albuminuria test	32135
	Quantification of total protein (urine)	32237

Software

All statistical analyses were conducted using the tidyverse,[4] comorbidity,[3] lme4,[5] and performance[6] packages of the R statistical Programming Language.[7]

Supplementary Tables

CKD stage	eGFR (in ml/min/1·73m²)	Referral recommended	Recommended times of monitoring (eGFR and proteinuria) per year	Approximated 5-year KF risk[#]
**1**	≥ 90	If proteinuria is severe*	1 (3 if proteinuria is severe)	<0.2%
**2**	60 – 89	If proteinuria is severe*	1 (3 if proteinuria is severe)	<0.2%
**3a**	45 – 59	If proteinuria is moderate*	1 (2 if proteinuria is moderate; 3 if proteinuria is severe)	i) 0.42%, ii) 2.42%
**3b**	30 – 44	If proteinuria is present*	2 (3 if proteinuria is severe)	i) 2.21%, ii) 12.24%
**4**	15 – 29	Always	3 (4+ if proteinuria is severe)	i) 11.2%, ii) 50.04%
**5**	< 15 or on KRT	Always	4+	i) 36.37%, ii) 92.86%

*Albuminuria is used as a surrogate for proteinuria; the threshold for referral varies between CKD stages; other reasons for referral may apply (e.g. CKD at a young age; uncertain etiology)

[#] using the 4-variable Kidney Failure Risk Equation[8] for a 70-year-old male from Northern America with albuminuria of i) 10 mg/g creatinine or ii) 500 mg/g creatinine. eGFR was set at 52 ml/min/1·73m² for CKD stage 3a, 37 ml/min/1·73m² for CKD stage 3b, 22 ml/min/1·73m² for CKD stage 4, 10 ml/min/1·73m² for CKD stage 5

Table S1. Overview of CKD stages and KDIGO recommendations for referral and monitoring. Adapted from Stevens et al.[S9] CKD = chronic kidney disease, eGFR = estimated glomerular filtration rate, KDIGO = Kidney Disease: Improving Global Outcomes, KF = kidney failure, KRT = kidney replacement therapy (dialysis or kidney transplantation)

CKD stage	3	4	5 without KRT	5 with KRT
**Total**	958,149	207,043	37,020	98,910
**Prevalence***	1·4%	0·3%	0·1%	0·1%
**Age in y, median (IQR)**	80 (72-85)	82 (73-86)	76 (65-84)	65 (54-76)
**Age brackets, count (%)**				
**0-30 years**	2,030 (0·2%)	752 (0·4%)	422 (1·1%)	3,466 (3·5%)
**31-50 years**	16,365 (1·7%)	4,140 (2·0%)	2,399 (6·5%)	15,257 (15·4%)
**51-70 years**	193,821 (20·2%)	34,343 (16·6%)	10,381 (28·0%)	42,831 (43·3%)
**71-85 years**	522,334 (54·5%)	106,938 (51·7%)	16,909 (45·7%)	30,964 (31·3%)
**86+ years**	223,599 (23·3%)	60,870 (29·4%)	6,909 (18·7%)	6,392 (6·5%)
**Female sex, count (%)**	520,639 (54·3%)	117,134 (56·6%)	17,618 (47·6%)	38,674 (39·1%)
**Nursing home inhabitant, count (%)**	70,362 (7·3%)	20,341 (9·8%)	2,857 (7·7%)	4,190 (4·2%)
**Palliative care in 2022, count (%)**	16,389 (1·7%)	5,789 (2·8%)	934 (2·5%)	1,352 (1·4%)
**Population density, decile median (IQR)**	5 (2-8)	5 (2-7)	5 (3-8)	5 (3-8)
**Deprivation index, decile median (IQR)**	6 (3-8)	6 (4-8)	6 (3-8)	6 (3-8)
**Nephrologist density, decile median (IQR)**	5 (3-7)	5 (3-7)	6 (3-8)	5 (3-8)
**Elixhauser index, median (IQR)**	6 (4-8)	7 (5-8)	6 (5-8)	6 (4-8)
**Diabetes mellitus, count (%)**	509,324 (53·2%)	121,754 (58·8%)	19,211 (51·9%)	41,784 (42·2%)
**Arterial hypertension, count (%)**	884,629 (92·3%)	197,594 (95·4%)	34,387 (92·9%)	93,318 (94·3%)

* Reference group: 69,050,132 patients from the general population who did not exit the statutory health care system in 2022/2023

Table S2. Prevalence of CKD stages 3-5 and baseline characteristics.
Binary variables are indicated with counts and % of the respective totals. Continuous variables are represented as medians with the interquartile range between the 25th and 75th percentile. CKD = chronic kidney disease, IQR = interquartile range, y = years

**Random effect**				**Intra-class coefficient**		
County				0·043		
State				0·013		
**Fixed effect**	**Unadjusted OR**	**CI**	**p-value**	**Adjusted OR**	**CI**	**p-value**
(Intercept)	-	-	-	(9·62)	(7·67 – 12·07)	(< 0·00001)
Female sex	0·65	0·64 – 0·66	< 0·00001	0·75	0·73 – 0·76	< 0·00001
Age per year	0·97	0·96 – 0·97	< 0·00001	0·96	0·96 – 0·96	< 0·00001
Nursing home inhabitant	0·50	0·49 – 0·52	< 0·00001	0·71	0·69 – 0·74	< 0·00001
Palliative care	0·63	0·60 – 0·67	< 0·00001	0·79	0·75 – 0·84	< 0·00001
Deprivation score, per decile	1·04	1·03 – 1·04	< 0·00001	1·02	1·00 – 1·04	0·091
Population density, per decile	1·01	1·00 – 1·01	< 0·00001	1·01	0·99 – 1·02	0·413
Nephrology density, per decile	1·01	1·01 – 1·01	< 0·00001	1·00	0·99 – 1·01	0·825
Congestive heart failure	1·02	1·00 – 1·03	0·079	0·85	0·83 – 0·87	< 0·00001
Cardiac arrythmia	1·02	1·00 – 1·04	0·069	1·02	0·99 – 1·04	0·134
Valvular disease	1·28	1·25 – 1·30	< 0·00001	1·19	1·16 – 1·22	< 0·00001
Pulmonary circulation disorder	1·22	1·18 – 1·26	< 0·00001	1·09	1·05 – 1·13	< 0·00001
Peripheral vascular disease	1·31	1·28 – 1·33	< 0·00001	1·14	1·11 – 1·16	< 0·00001
Hypertension without complications	0·46	0·45 – 0·47	< 0·00001	1·97	1·88 – 2·07	< 0·00001
Hypertension with complications	2·70	2·65 – 2·76	< 0·00001	5·05	4·80 – 5·31	< 0·00001
Paralysis	0·85	0·82 – 0·89	< 0·00001	0·85	0·81 – 0·89	< 0·00001
Other neurological disorders	0·90	0·87 – 0·92	< 0·00001	0·96	0·93 – 1·00	0·045
Chronic pulmonary disease	1·15	1·12 – 1·17	< 0·00001	1·03	1·00 – 1·05	0·025
Diabetes without chronic complications	0·81	0·79 – 0·83	< 0·00001	0·85	0·83 – 0·88	< 0·00001
Diabetes with chronic complications	1·17	1·15 – 1·19	< 0·00001	1·03	1·01 – 1·06	0·00417
Hypothyroidism	1·15	1·13 – 1·18	< 0·00001	1·16	1·13 – 1·19	< 0·00001
Liver disease	1·21	1·18 – 1·23	< 0·00001	0·99	0·97 – 1·02	0·500
Peptic ulcer disease	1·16	1·09 – 1·23	< 0·00001	1·01	0·94 – 1·08	0·843
Human immunodeficiency virus	1·30	0·97 – 1·76	0·084	0·98	0·71 – 1·35	0·892
Lymphoma	1·31	1·23 – 1·40	< 0·00001	1·23	1·14 – 1·32	< 0·00001
Metastatic cancer	1·15	1·10 – 1·20	< 0·00001	1·19	1·13 – 1·25	< 0·00001
Solid tumor without metastasis	1·26	1·23 – 1·29	< 0·00001	1·25	1·22 – 1·28	< 0·00001
Rheumatoid arthritis	1·18	1·14 – 1·21	< 0·00001	1·14	1·10 – 1·17	< 0·00001
Coagulopathy	1·26	1·21 – 1·30	< 0·00001	1·09	1·05 – 1·14	0·00001
Obesity	1·39	1·37 – 1·42	< 0·00001	1·12	1·09 – 1·14	< 0·00001
Weight loss	0·94	0·90 – 0·98	0·006	0·94	0·89 – 0·99	0·015
Fluid and electrolyte disorders	3·12	3·04 – 3·20	< 0·00001	3·07	2·98 – 3·15	< 0·00001
Blood loss anemia	1·31	1·22 – 1·41	< 0·00001	1·11	1·02 – 1·20	0·012
Anemia deficiency	1·61	1·57 – 1·65	< 0·00001	1·47	1·43 – 1·51	< 0·00001
Alcohol abuse	1·08	1·03 – 1·14	0·002	0·78	0·74 – 0·83	< 0·00001
Drug abuse	0·85	0·79 – 0·91	0·00002	0·90	0·83 – 0·97	0·010

Psychosis	0·74	0·69 – 0·79	< 0·00001	0·86	0·80 – 0·93	0·00007
Depression	0·91	0·89 – 0·93	< 0·00001	0·93	0·91 – 0·95	< 0·00001

Table S3. Sensitivity analysis of table 3 including all individual comorbidities of the Elixhauser index. A multi-variable mixed logistic regression model was used to evaluate patients for nephrologist referral in 2022. Referred patients had at least one health claim by a nephrologist. The intra-class coefficient (ICC) measures the proportion of total variance attributable to county and state levels, with values closer to 1 indicating more variation between regions and values closer to 0 indicating more variation between individuals. Female sex, nursing home inhabitant, and palliative care were binary variables; age was continuous. Deprivation score, population density, and nephrologist density were categorized into deciles to assess their impact across the distribution of the German population, with higher deciles indicating higher density or deprivation. Individual comorbidities of the Elixhauser index were included as separate, binary variables in the model. *n*(total) = 207,043; *n*(endpoint) = 134,143. CKD = chronic kidney disease, CI = confidence interval, OR = odds ratio

Random effect				Intra-class coefficient		
County				0·029		
State				0·007		
**Fixed effect**	**Unadjusted OR**	**CI**	**p-value**	**Adjusted OR**	**CI**	**p-value**
(Intercept)	-	-	-	(2·42)	(2·06 – 2·83)	(< 0·00001)
Female sex	0·78	0·77 – 0·79	< 0·00001	0·84	0·83 – 0·84	< 0·00001
Age per year	0·97	0·97 – 0·98	< 0·00001	0·97	0·97 – 0·97	< 0·00001
Nursing home inhabitant	1·04	1·02 – 1·05	0·00001	1·25	1·23 – 1·27	< 0·00001
Palliative care	1·04	1·01 – 1·08	0·009	1·09	1·06 – 1·13	< 0·00001
Deprivation score, per decile	1·03	1·03 – 1·03	< 0·00001	1·02	1·00 – 1·03	0·046
Population density, per decile	1·02	1·02 – 1·02	< 0·00001	1·01	1·00 – 1·02	0·130
Nephrology density, per decile	1·02	1·01 – 1·02	< 0·00001	1·00	0·99 – 1·01	0·544
Elixhauser score, per point	1·11	1·11 – 1·11	< 0·00001	1·13	1·13 – 1·14	< 0·00001

Table S4. Determinants of nephrology referral in CKD stage 3.
A multi-variable mixed logistic regression model was used to evaluate nephrologist referral of patients with CKD stage 3 in 2022. Referred patients had at least one health claim by a nephrologist. The intra-class coefficient (ICC) measures the proportion of total variance attributable to county and state levels, with values closer to 1 indicating more variation between regions and values closer to 0 indicating more variation between individuals. Female sex, nursing home inhabitant, and palliative care were binary variables; age was continuous. Deprivation score, population density, and nephrologist density were categorized into deciles to assess their impact across the distribution of the German population, with higher deciles indicating higher density or deprivation. Higher Elixhauser scores indicated greater comorbidity. ***n***(total) = 958,149; ***n***(endpoint) = 344,358. CKD = chronic kidney disease, CI = confidence interval, OR = odds ratio

Random effect				Intra-class coefficient		
County					0·118	
State					0·025	
**Fixed effect**	**Unadjusted exponential estimate**	**CI**	**p-value**	**Adjusted exponential estimate**	**CI**	**p-value**
(Intercept)	-	-	-	(11·66)	(7·83 – 17·36)	(< 0·00001)
Female sex	0·78	0·75 – 0·82	< 0·00001	0·87	0·82 – 0·91	< 0·00001
Age per year	0·98	0·97 – 0·98	< 0·00001	0·97	0·97 – 0·98	< 0·00001
Nursing home inhabitant	0·62	0·58 – 0·68	< 0·00001	0·70	0·64 – 0·77	< 0·00001
Palliative care	0·77	0·67 – 0·88	0·00021	0·91	0·78 – 1·07	0·254
Deprivation score, per decile	0·97	0·96 – 0·98	< 0·00001	1·01	0·98 – 1·05	0·476
Population density, per decile	1·00	1·00 – 1·01	0·320	0·97	0·94 – 1·00	0·093
Nephrology density, per decile	0·96	0·96 – 0·97	< 0·00001	0·99	0·96 – 1·02	0·384
Elixhauser score, per point	1·10	1·09 – 1·11	< 0·00001	1·17	1·15 – 1·18	< 0·00001

Table S5. Determinants of nephrology referral in CKD stage 5 without KRT.
A multi-variable mixed logistic regression model was used to evaluate nephrologist referral of patients with CKD stage 5 without KRT in 2022. Referred patients had at least one health claim by a nephrologist. The intra-class coefficient (ICC) measures the proportion of total variance attributable to county and state levels, with values closer to 1 indicating more variation between regions and values closer to 0 indicating more variation between individuals. Female sex, nursing home inhabitant, and palliative care were binary variables; age was continuous. Deprivation score, population density, and nephrologist density were categorized into deciles to assess their impact across the distribution of the German population, with higher deciles indicating higher density or deprivation. Higher Elixhauser scores indicated greater comorbidity. *n*(total) = 37,020; *n*(endpoint) = 27,617. CKD = chronic kidney disease, CI = confidence interval, KRT = kidney replacement therapy, OR = odds ratio

**n (total) = 160,965**					
**Age in years**	**0-30**	**31-50**	**51-70**	**71-85**	**+86**
**Non-referred (n = 59,389)**	81	604	7,221	27,229	24,254
**Referred (n = 101,576)**	456	2,450	18,646	55,068	24,956
2-year risk of ambulatory dialysis (uncensored)					
**Non-referred**	0 (0·0%)	NA*	104 (1·4%)	192 (0·7%)	35 (0·1%)
**Referred**	65 (14·3%)	285 (11·6%)	1,354 (7·3%)	2,181 (4·0%)	364 (1·5%)
2-year risk of ambulatory palliative care (uncensored)					
**Non-referred**	NA*	NA*	163 (2·3%)	1,374 (5·0%)	2,604 (10·7%)
**Referred**	NA*	NA*	322 (1·7%)	2,218 (4·0%)	2,154 (8·6%)

* Due to anonymization, data has been blurred for groups containing 1-30 patients to protect privacy

Table S6. 2-year incidence of ambulatory dialysis and ambulatory palliative care among prevalent CKD stage 4 patients.

Patients who had a CKD stage 4 diagnosis in the first quarter of 2022 and a reconfirmation of this diagnosis in another quarter in 2022 were included in this analysis. As opposed to the other analyses in this study, this supplementary analysis also included patients who exited the statutory health care system in 2022 or 2023. The table reports the uncensored 2-year risk of receiving ambulatory dialysis or ambulatory palliative care by age group and referral status. Both endpoints were handled as binary variables. The 2-year observation period started in the first quarter of 2022 and ended in the last quarter of 2023. Data on performance of dialysis or palliative care in the hospital setting were unavailable. NA = not available

Supplementary Figures

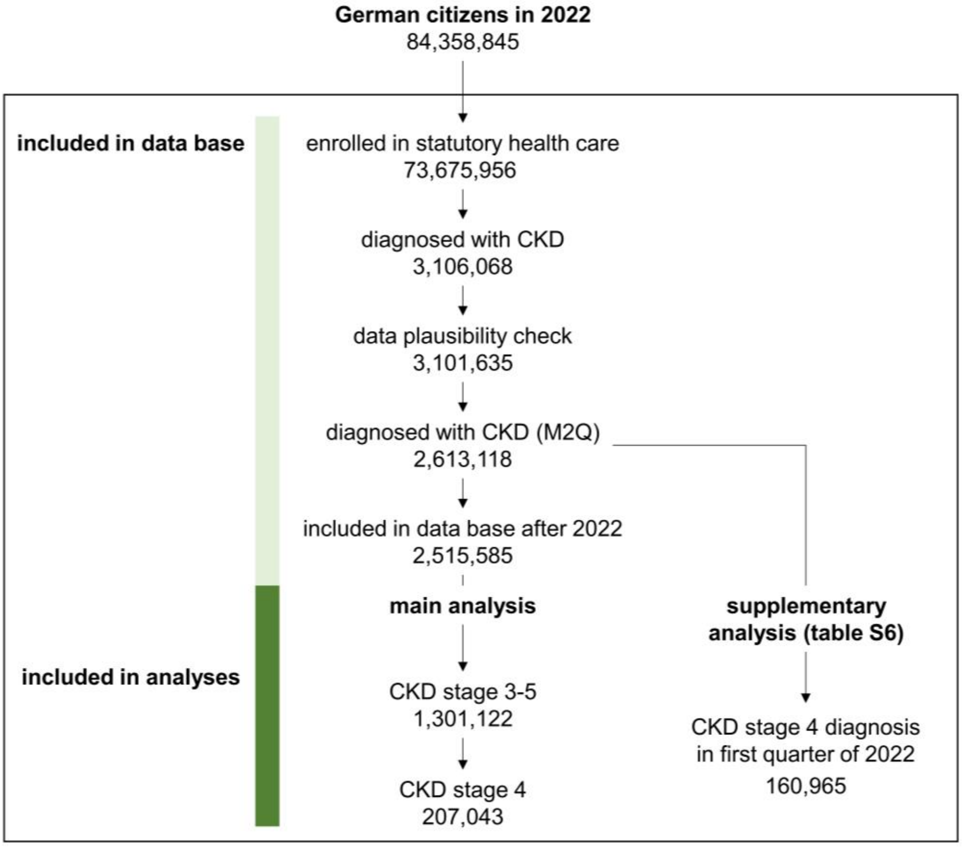

Figure S1. Flowchart of the inclusion of patients.
All patients with statutory health care in the year 2022 were considered for the analyses, including children. We identified all patients with a diagnosis of CKD or dialysis in at least 1 quarter of 2022 and performed a data plausibility check, excluding patients >125 years old or those without entries for the variables of biological sex and residence. In the main analyses, patients with reconfirmed CKD according to the M2Q criteria (see Supplementary Material) were included in the analyses if they were followed-up by any physician in the German health care system in 2022Q4 or in 2023, excluding those who had exited the statutory health care system for any reason in 2022. In a supplementary analysis assessing incidences of dialysis and palliative care, patients with CKD stage 4 in the first quarter of 2022 and reconfirmation of this diagnosis in an additional quarter of 2022 (M2Q) were included, regardless of whether they had exited the statutory health care system. CKD = chronic kidney disease

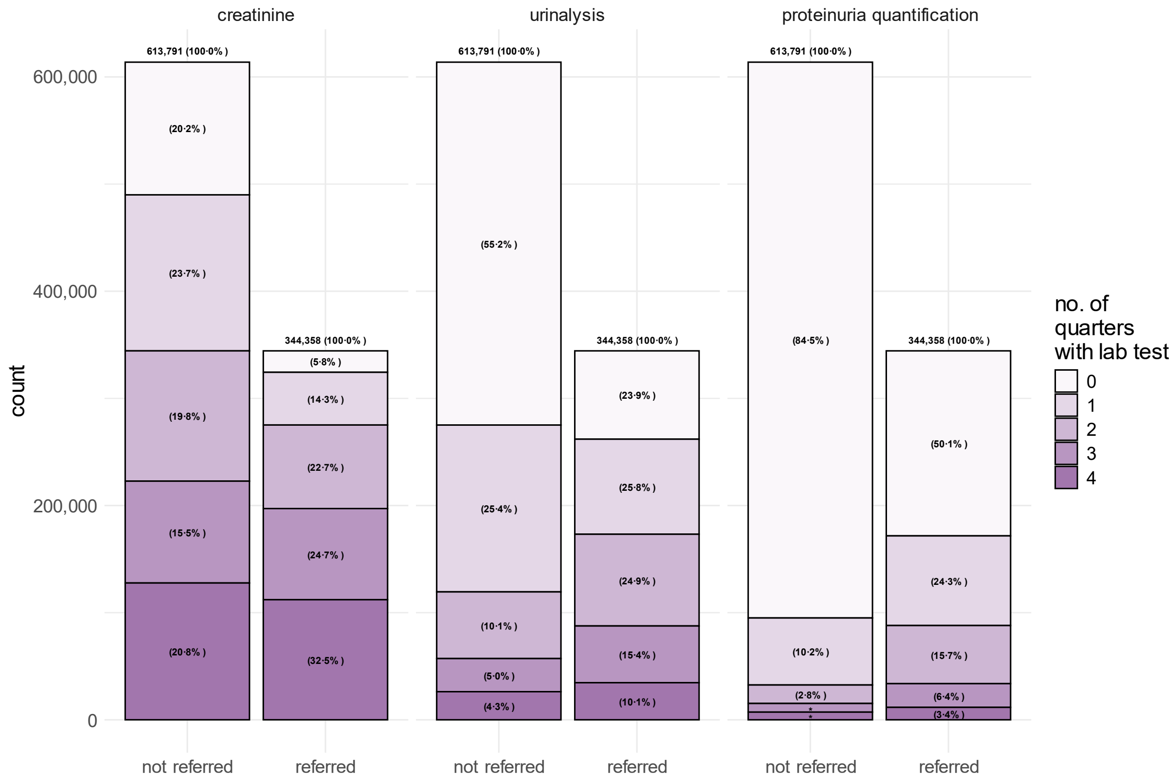

Figure S2. Frequency of serum creatinine and proteinuria monitoring in patients with CKD stage 3, comparing those referred and not referred to a nephrologist.

The figure shows the number of quarters in 2022 during which serum creatinine was measured, any type of urinalysis was conducted, and total proteinuria was quantified. Patients are grouped by referral status, with referral defined as having at least one ambulatory health claim from a nephrologist in 2022. Percentages (%) represent the proportion of patients in each referral group, relative to the total number of patients in that group. *n*(total) = 958,149. CKD = chronic kidney disease

***** proteinuria quantification, not referred: 3 quarters (1·3%), 4 quarters (1·2%)

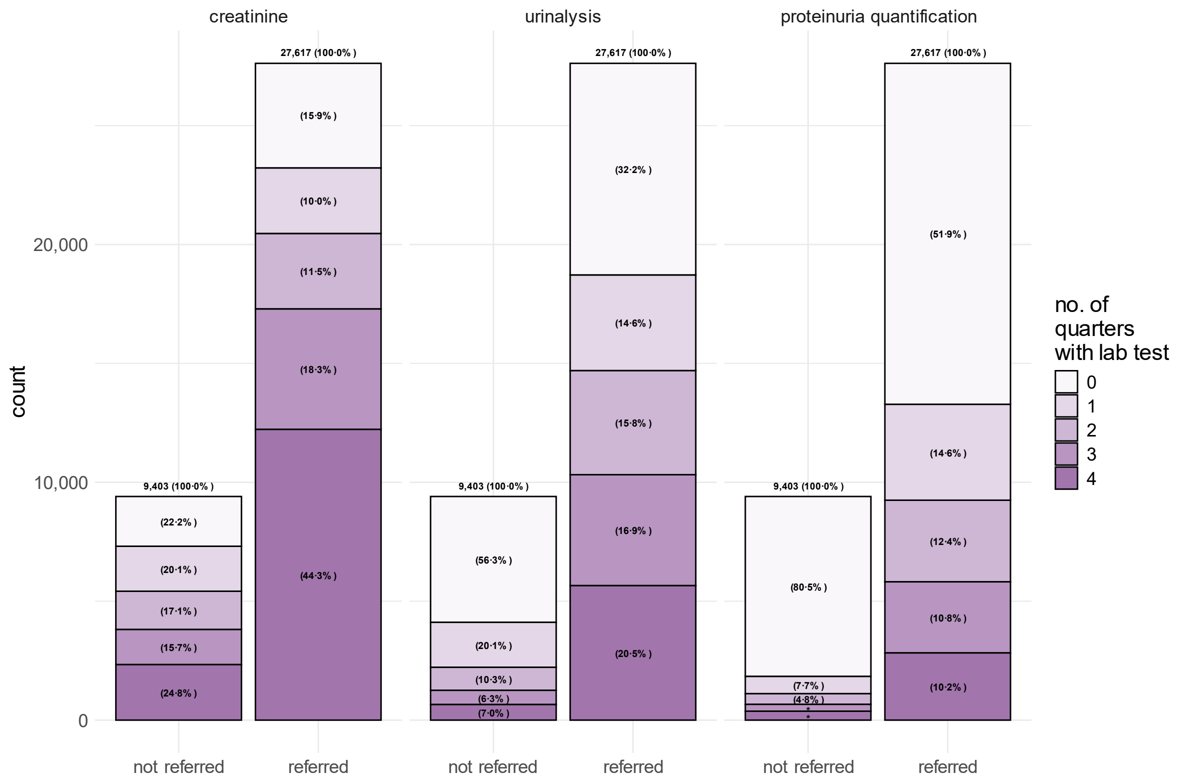

Figure S3. Frequency of serum creatinine and proteinuria monitoring in patients with CKD stage 5 without KRT, comparing those referred and not referred to a nephrologist.

The figure shows the number of quarters in 2022 during which serum creatinine was measured, any type of urinalysis was conducted, and total proteinuria was quantified. Patients are grouped by referral status, with referral defined as having at least one ambulatory health claim from a nephrologist in 2022. Percentages (%) represent the proportion of patients in each referral group, relative to the total number of patients in that group. *n*(total) = 37,020. CKD = chronic kidney disease, KRT = kidney replacement therapy

***** proteinuria quantification, not referred: 3 quarters (3·1%), 4 quarters (4·0%)

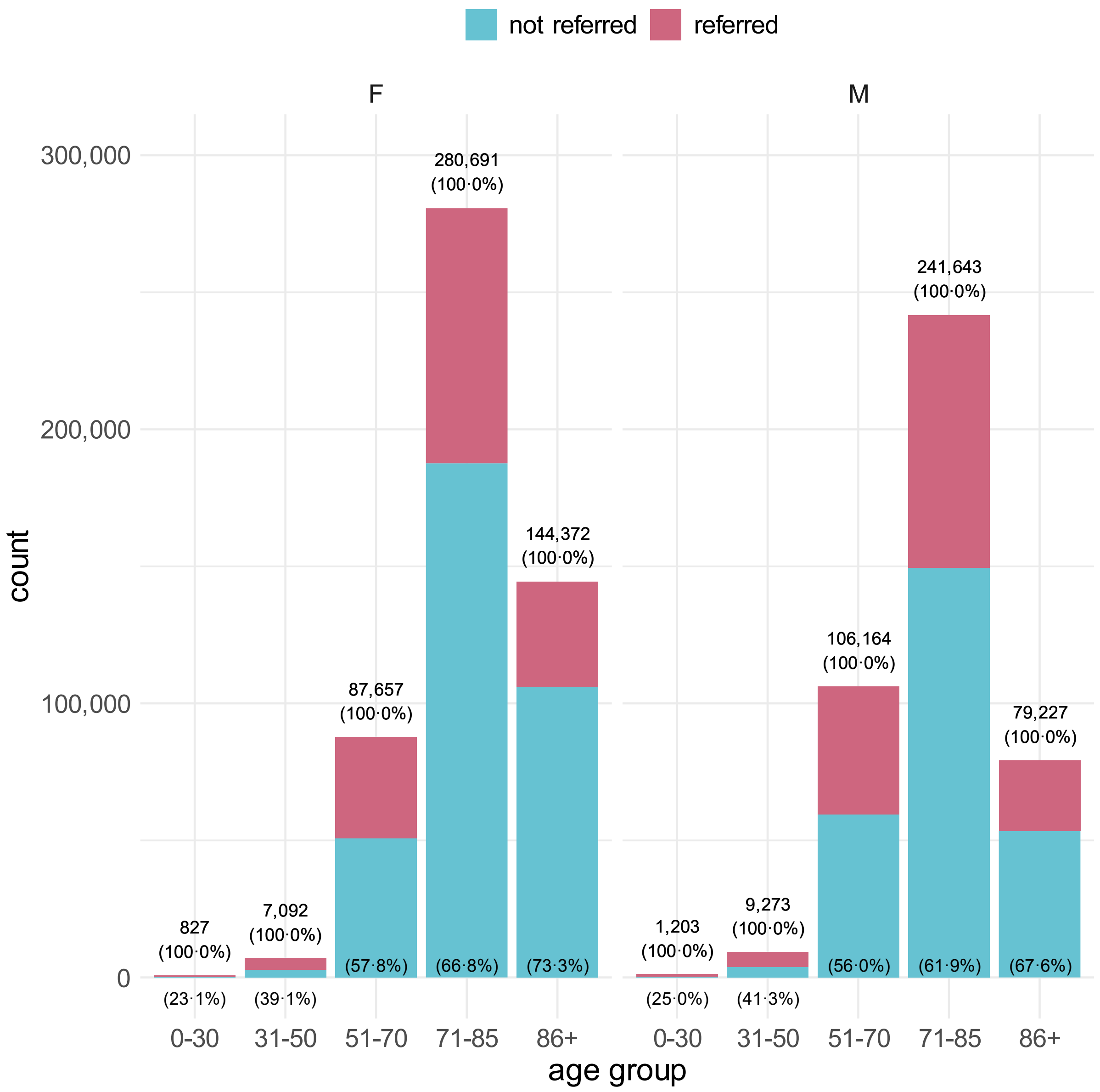

Figure S4. Age-dependent referral patterns for males and females with CKD stage 3 to nephrology services. Patients were stratified by biological sex (male and female) and grouped by age ranges. Referral was defined by the presence of at least one ambulatory health claim from a nephrologist in 2022. The figure displays the percentage of non-referred patients in each age group, with the remaining percentage (not explicitly shown) representing referred patients. The sum of these proportions equals 100% for each age group. *n*(total) = 958,149. CKD = chronic kidney disease, F = female, M = male

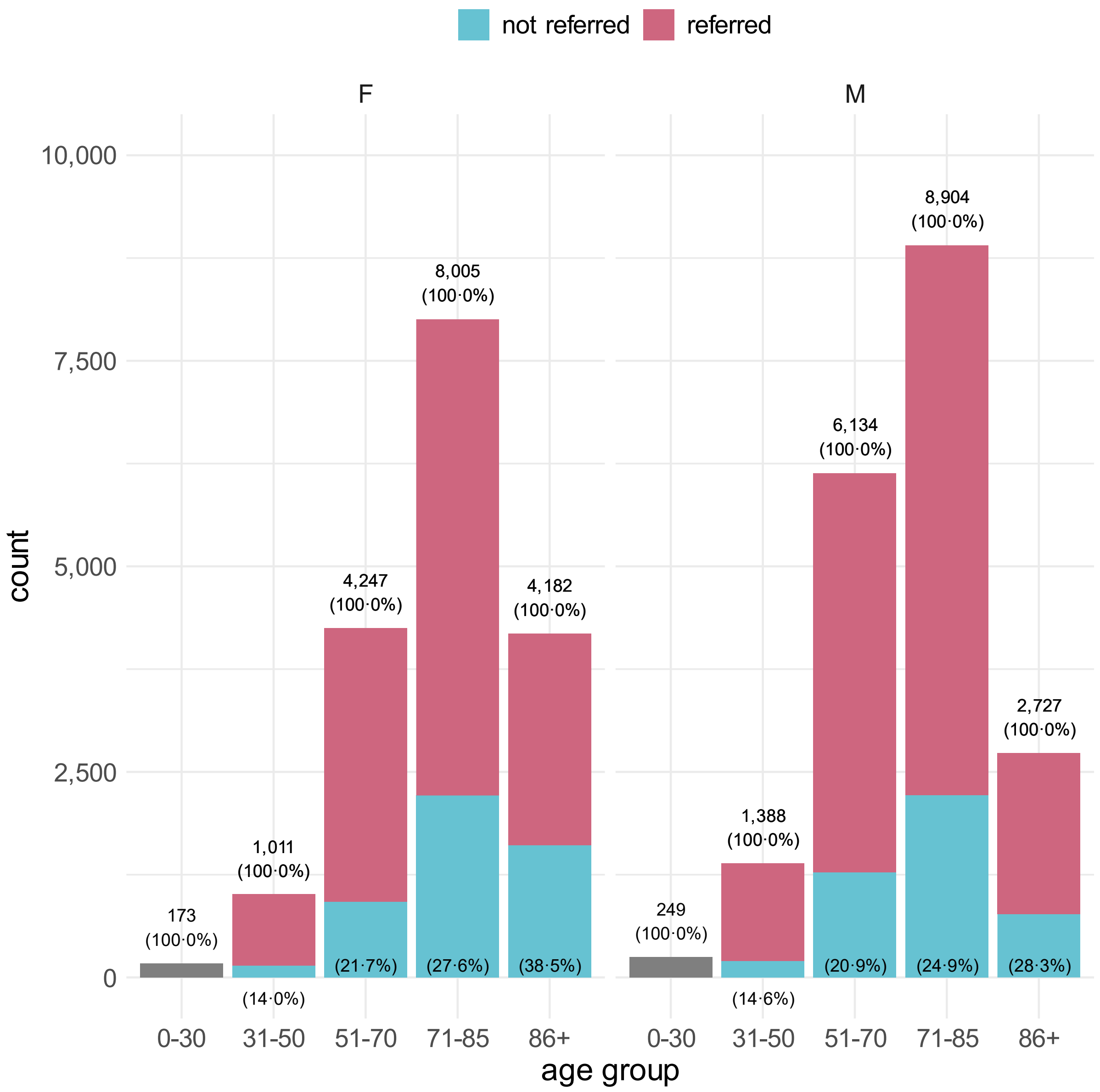

Figure S5. Age-dependent referral patterns for males and females with CKD stage 5 without KRT to nephrology services.

Patients were stratified by biological sex (male and female) and grouped by age ranges. Referral was defined by the presence of at least one ambulatory health claim from a nephrologist in 2022. The figure displays the percentage of non-referred patients in each age group, with the remaining percentage (not explicitly shown) representing referred patients. The sum of these proportions equals 100% for each age group. Because of anonymization regulations and very low case numbers in the age group 0-30, it was not possible to further differentiate between referred and non-referred patients in this group (grey bars). *n*(total) = 37,020. CKD = chronic kidney disease, F = female, KRT = kidney replacement therapy, M = male

Supplementary References

S1. Bundesvereinigung K. Fachgruppenzuordnung. 2024. https://applications.kbv.de/S_BAR2_FACHGRUPPENZUORDNUNG_V1.02.xhtml (accessed 11 Sep 2024.

S2. Quan H, Sundararajan V, Halfon P, et al. Coding algorithms for defining comorbidities in ICD-9-CM and ICD-10 administrative data. *Med Care* 2005; **43**(11): 1130-9.

S3. Gasparini A. Comorbidity: An R package for computing comorbidity scores. *J Open Source Softw* 2018; **3**(23): 648.

S4. Wickham H, Averick M, Bryan J, et al. Welcome to Tidyverse. *J Open Source Softw* 2019.

S5. Bates D, Maechler M, Bolker B, et al. Fitting Linear Mixed-Effects Models Using lme4. *J Stat Softw* 2015; **67**(1): 1-48.

S6. Lüdecke D, Ben-Sachar MS, Patil I, Waggoner P, Makowski D. Performance: An R Package for Assessment, Comparison and Testing of Statistical Models. *J Open Source Softw* 2021; **6**(60): 3139.

S7. R Core Team. R: A Language and Environment for Statistical Computing. *R Foundation for Statistical Computing, Vienna, Austria* 2021.

S8. Tangri N, Grams ME, Levey AS, et al. Multinational Assessment of Accuracy of Equations for Predicting Risk of Kidney Failure: A Meta-analysis. *JAMA* 2016; **315**(2): 164-74.

S9. Stevens PE, Ahmed SB, Carrero JJ, et al. KDIGO 2024 Clinical Practice Guideline for the Evaluation and Management of Chronic Kidney Disease. *Kidney Int* 2024; **105**(4): S117-S314.
